# Multivalent protein–protein interactions are pivotal regulators of eukaryotic Hsp70 complexes

**DOI:** 10.1007/s12192-022-01281-1

**Published:** 2022-06-07

**Authors:** Oleta T. Johnson, Jason E. Gestwicki

**Affiliations:** Department of Pharmaceutical Chemistry and the Institute for Neurodegenerative Diseases, University of California San Francisco, San Francisco, CA 94158 USA

**Keywords:** J-domain protein, Nucleotide exchange factor, Hsp110, Bag domain, Protein folding, Protein aggregation

## Abstract

Heat shock protein 70 (Hsp70) is a molecular chaperone and central regulator of protein homeostasis (proteostasis). Paramount to this role is Hsp70’s binding to client proteins and co-chaperones to produce distinct complexes, such that understanding the protein–protein interactions (PPIs) of Hsp70 is foundational to describing its function and dysfunction in disease. Mounting evidence suggests that these PPIs include both “canonical” interactions, which are universally conserved, and “non-canonical” (or “secondary”) contacts that seem to have emerged in eukaryotes. These two categories of interactions involve discrete binding surfaces, such that some clients and co-chaperones engage Hsp70 with at least two points of contact. While the contributions of canonical interactions to chaperone function are becoming increasingly clear, it can be challenging to deconvolute the roles of secondary interactions. Here, we review what is known about non-canonical contacts and highlight examples where their contributions have been parsed, giving rise to a model in which Hsp70’s secondary contacts are not simply sites of additional avidity but are necessary and sufficient to impart unique functions. From this perspective, we propose that further exploration of non-canonical contacts will generate important insights into the evolution of Hsp70 systems and inspire new approaches for developing small molecules that tune Hsp70-mediated proteostasis.

## Introduction

In order to maintain protein homeostasis (proteostasis), every cell employs a coordinated network of molecular chaperones that ensures the exquisite balance of protein levels. These chaperone networks are frequently dysregulated in diseases, including cancer, neurodegeneration, and viral infection, underscoring their importance (Labbadia and Morimoto 2015). Central components of this network are members of the heat shock protein 70 (Hsp70) family, which regulate protein quality control in organisms—from bacteria to humans—by binding to unfolded, misfolded, or damaged protein substrates, termed “clients.” Hsp70s then deliver clients to downstream pathways, including those involved in folding, trafficking, or degradation (M. P. Mayer and Bukau 2005). How do Hsp70s choose between these dramatically different outcomes? Why do they promote folding of a client under some conditions but turnover under others? These questions are at the center of many ongoing studies in the field. Moreover, Hsp70s need to make these decisions for an impressive variety of proteins because they recognize a simple motif enriched in hydrophobic amino acids (Gragerov et al. 1994; Rudiger 1997) that is common throughout the proteome (Srinivasan et al. 2012; Behnke et al. 2016), such that most proteins are likely to be clients under the right circumstances (Calloni et al. 2012). Thus, understanding the mechanisms of Hsp70-mediated quality control might provide significant insight into regulation of the proteome and how it might be leveraged to treat disease.

**Fig. 1:**
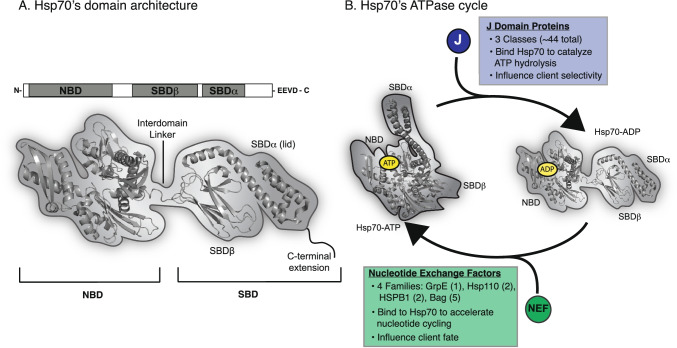
Members of the Hsp70 family have a conserved architecture and ATPase cycle. **A** General structure of Hsp70 family members, including a nucleotide-binding domain (NBD), substrate-binding domain (SBD). The SBD is sub-divided into SBDβ, SBDα (lid) and C-terminal extension. The various Hsp70 orthologs vary in the length and composition of the C-terminal region and cytoplasmic isoforms of eukaryotic Hsp70s also have an EEVD motif. The structure of the prokaryotic Hsp70, DnaK (PDB 2KHO), is shown, along with a cartoon representation. **B** Schematic of the ATPase cycle of Hsp70s (PDB 2KHO and 5NRO), highlighting the conformational changes that accompany hydrolysis and the roles of the co-chaperones: J-domain proteins (JDPs) and nucleotide exchange factors (NEFs)

Across organisms, members of the Hsp70 family exhibit remarkable conservation of molecular structure. All orthologs consist of two domains: a nucleotide-binding domain (NBD) and a substrate-binding domain (SBD) (Matthias P. Mayer and Gierasch 2019). The SBD is further sub-divided into three regions: (i) a beta-barrel domain (SBDβ), a (ii) an alpha-helical lid (SBDα or lid), and (iii) a disordered C-terminal extension (Fig. 1A) (Matthias P. Mayer and Gierasch 2019). Pioneering studies of the bacterial Hsp70, DnaK, have provided invaluable insight into the structure of these regions and how their intramolecular motions are coordinated. Specifically, the NBD contains a cleft that binds and hydrolyzes ATP (McCarty et al. 1995), resulting in dramatic, long-range conformational changes throughout the NBD, SBDβ, and lid (Fig. 1B) (A. Buchberger et al. 1994; Alexander Buchberger et al. 1995; Pellecchia et al. 2000; J. Jiang et al. 2005; Meng et al. 2018; Zuiderweg et al. 2017; English et al. 2017; Lai et al. 2017; Meng et al. 2018; Wu et al. 2020). For example, in the ATP-bound “open” state, the lid is docked to the NBD, leaving the SBDβ available to weakly interact with clients (Arhar et al. 2021; Qi et al. 2013); then, upon hydrolysis, the ADP-bound state undergoes a significant conformational change and the lid docks to the SBDβ to form a “closed” state that binds clients with stronger affinity (Schlecht et al. 2011; Rüdiger et al. 1997; Matthias P Mayer et al. 2000; Bertelsen et al. 2009; Zhuravleva and Gierasch 2015). Consistent with its high conservation, Hsp70s from other organisms seem to undergo similar (but not identical) conformational changes and to interact with client proteins in a way that is broadly comparable to DnaK (Meng et al. 2018; Wu et al. 2020).

While these conformational changes are important for Hsp70’s interactions with clients, this activity does not fully describe their decision-making ability. Rather, the diverse functions of Hsp70 require coordination with a suite of co-chaperone proteins that tune its activity and program client fate. In humans, these co-chaperones include three major families: the J-domain proteins (JDPs), the nucleotide exchange factors (NEFs), and the tetratricopeptide repeats (TPR) proteins. These co-chaperones make direct physical contact with Hsp70s (Zuiderweg et al. 2017) and a subset of them, JDPs and NEFs, accelerate nucleotide cycling (Fig. 1B) (Rosenzweig et al. 2019; Kampinga and Craig 2010). Moreover, many of these co-chaperones also serve as adapters, which physically bridge Hsp70s and their clients with the pathways involved in folding, trafficking, degradation, and other outcomes (Rosenzweig et al. 2019). Thus, Hsp70s are only functional when they are part of a multi-protein complex, in which the chaperone, its clients and its co-chaperones engage in protein–protein interactions (PPIs) (Schröder et al. 1993). Notably, most eukaryotic cells express multiple members of each chaperone and co-chaperone family; for example, there are at least 11 distinct Hsp70 isoforms (Tavaria et al. 1996) and ~ 45 JDP genes in humans (Kampinga and Craig 2010). This diversity allows Hsp70s to form many possible combinations of complexes to diversify its functions. Moreover, these various complexes are dynamic—they form and dissolve in response to temperature (Palleros et al. 1991), ATP cycling (Palleros et al. 1991; Matthias P. Mayer and Gierasch 2019), competing PPIs (Rosam et al. 2018; Gowda et al. 2018; Johnson et al. 2022), post-translational modifications (PTMs) (Assimon et al. 2015; Nitika et al. 2020), and the thermodynamic and kinetic requirements of the client itself (Sekhar et al. 2012).

Because Hsp70s act as part of dynamic, multi-protein complexes, one key way to understanding their function is to probe their PPIs. Indeed, decades of work has revealed how clients and co-chaperones interact with Hsp70s. For example, JDPs use a J-domain to contact the NBD and interdomain linker (Kampinga and Craig 2010) and NEFs use a variety of domains to bind surfaces on the NBD (Bracher and Verghese 2015). Collectively, we refer to these PPIs as the “canonical” interactions to denote their importance in the history of Hsp70 research and their striking conservation in both prokaryotes and eukaryotes. While the canonical PPIs of Hsp70 are sufficient for some functions, such as promoting nucleotide cycling, there has been an apparent expansion of PPIs in eukaryotes to add new functions. For example, TPR co-chaperones, which are not present in prokaryotes, use their TPR domains to bind an EEVD motif at the C-terminus of cytosolic, eukaryotic Hsp70s—serving as adapters that link cytosolic Hsp70s to cellular pathways involved in folding, localization, and degradation (D’Andrea 2003; Weber et al. 2020). Evidence suggests that eukaryotic Hsp70s have also evolved to engage in additional contacts with the clients, JDPs, and NEFs that already contain canonical binding domains. These secondary interactions involve interfaces that are distinct from those employed in canonical binding, such that Hsp70s can interact with some clients or co-chaperones via multiple points of contact. In many biological systems, it is well known that a mixture of primary and secondary binding sites stabilizes complexes through enhanced avidity (Kitov and Bundle 2003; Errington et al. 2019); thus, one role of multivalent, non-canonical contacts in the eukaryotic Hsp70 systems might be to likewise tune avidity. However, this effect has rarely been explored or quantified for Hsp70 PPIs and the contributions of individual contacts to avidity remain unclear. In contrast, it is becoming apparent that these secondary contacts are necessary and sufficient for many of the ascribed functionalities of eukaryotic Hsp70 sub-networks.

To complement recent reviews on Hsp70 structure and function (Freilich et al. 2018; Clerico et al. 2015; Matthias P. Mayer and Gierasch 2019; Lang et al. 2021), we focus here on discussing what is known about non-canonical contacts. We chose this focus because non-canonical interactions seem to be often overlooked—perhaps because the prokaryotic models lack them. From this literature analysis, we propose that a deeper understanding of non-canonical interactions is critical to revealing the logic of Hsp70-mediated proteostasis in eukaryotes. function.

### Hsp70 clients

An integral part of Hsp70 function is its ability to bind client proteins and promote their native folding (Sekhar et al. 2012) and disaggregation (Nillegoda et al. 2018; Melo et al. 2022). Initial efforts to identify what sequences are binding sites for Hsp70s focused on screening peptide libraries (Gragerov et al. 1994; Gragerov and Gottesman 1994; Rudiger 1997; Behnke et al. 2016). Broadly, these studies reveal a preference of Hsp70s for sequences of 5 to 7 residues enriched in hydrophobic and non-polar amino acids (Blond-Elguindi 1993; Gragerov et al. 1994; Richarme and Kohiyama 1993; Rudiger 1997). This motif makes logical sense, as hydrophobic sequences are normally sequestered into the interior of properly folded proteins; thus, by binding to these motifs, Hsp70s might discriminate between folded and misfolded/unfolded clients. Guided by these large-scale peptide array studies, algorithms have been created to computationally predict Hsp70 binding sites in client amino acid sequences. Most recently, approaches based on a position-specific scoring matrix (PSSM) (Van Durme et al. 2009; Schneider et al. 2016; Nordquist et al. 2021) and position-independent scoring matrix (PISM) (Gutierres et al. 2019) have been reported. Here, we will briefly review what is known about the structure of canonical client interactions before turning our attention to the non-canonical client contacts present in eukaryotes.

#### Canonical Hsp70/client interactions

**Fig. 2:**
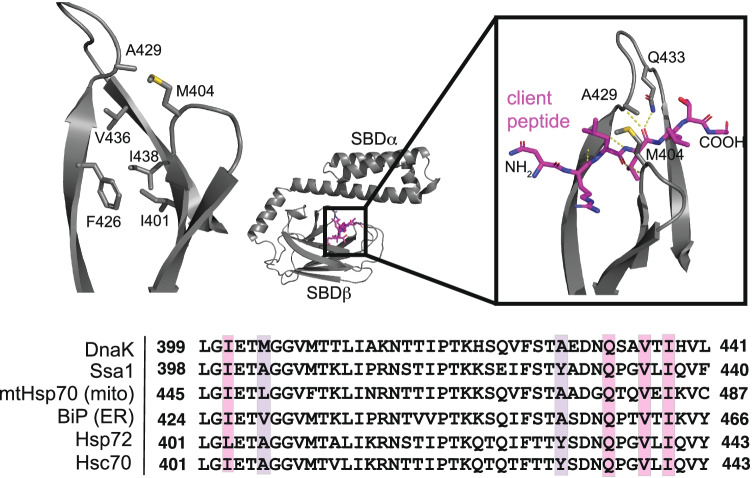
Canonical clients bind a conserved, hydrophobic groove in Hsp70’s SBDβ. The SBDβ and lid of DnaK is pictured bound to the model client peptide NRLLLTG (PDB 1DKZ), highlighting the key residues involved. The conservation of those residues across Hsp70 orthologs in *E. coli* (DnaK), yeast (Ssa1), and humans (mtHsp70, BiP, Hsp72, and Hsc70) is shown from a CLUSTALW multiple sequence alignment

Early work with DnaK showed that a canonical peptide motif binds in a linear, extended conformation within a hydrophobic groove of SBDβ (Fig. 2) (Landry et al. 1992; Swain et al. 2006; Marcinowski et al. 2013; Schlecht et al. 2011). The SBDβ is composed of an 8-stranded, antiparallel beta barrel (Matthias P Mayer et al. 2000; Zhu et al. 1996), and conserved residues within its groove, namely Ile401, Phe426, Val436, and Ile438, are critical for binding to a model peptide (NRLLLTG) (Fig. 2) (Zhu et al. 1996; Larkin et al. 2007). Additionally, the Gln433 side chain, along with the backbone at Met404 and Ala429, form hydrogen bonds with the backbone of NRLLLTG, consistent with the broad selectivity of Hsp70s for non-polar sequences with minor contributions from side chain recognition. All three of these residues are in conformationally dynamic β-loops of the SBD that form an arch over the hydrophobic cleft and appear to moderate client association (Fig. 2) (Zhu et al. 1996; Stevens et al. 2009). The relatively shallow nature of the groove is also consistent with the weak measured affinity for Hsp70’s clients, which is measured to be ~ 0.1 to 10 μM depending on the sequence (McCarty et al. 1996). It should be noted that Met404 and Ala429 are more variable across Hsp70 homologs (Fig. 2) and appear to account for differences in client selectivity within Hsp70 family members (Rüdiger et al. 2000; Marcinowski et al. 2011). Nevertheless, the overall theme is that Hsp70s use their conserved SBD to bind exposed, hydrophobic motifs in clients.

#### Non-canonical Hsp70/client interactions

Recently, Hsp70s were found to interact with sequences that do not fit the normal consensus for a canonical client (Sarkar et al. 2008; Burmann et al. 2020; Tao et al. 2021), often containing negative charges or other polar groups. Concurrently, evidence has emerged that Hsp70s are capable of binding to clients that are in an assortment of non-linear conformational states (Mashaghi et al. 2016), and not just linear, extended motifs. Together, these types of observations suggest that eukaryotic Hsp70s may have evolved alternative ways of binding clients. Intriguingly, two intrinsically disordered proteins (IDPs), microtubule-associated protein tau (tau) and alpha-synuclein (α-syn), have been shown to contain both canonical and non-canonical interaction motifs (Sarkar et al. 2008; Taylor et al. 2018; Burmann et al. 2020), and, thus, have become interesting models for probing the two modes of client binding.

**Fig. 3:**
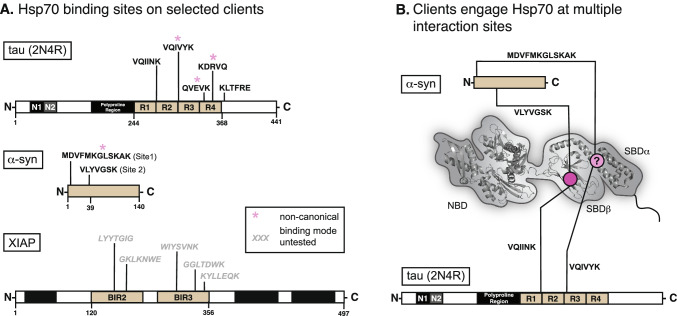
Certain clients engage Hsp70 both canonically and non-canonically. **A** The domain architecture of three client proteins is shown, with known Hsp70-binding sequences displayed. A subset of these sequences is experimentally shown to bind in a nucleotide-independent, non-canonical manner and they are not competitive with canonical, model substrates for binding SBDβ. **B** Schematic to illustrate that some clients, including α-syn and tau, have both canonical and non-canonical binding motifs, and are able to interact with both the SBDβ and SBDα domains of Hsp70s. Still, the exact binding site of non-canonical peptides is unknown. See text for citations and details

In a class of neurodegenerative diseases, termed tauopathies, tau accumulates to form toxic aggregates (Mandelkow and Mandelkow 2012). In these disorders, cytosolic Hsp70s have been closely associated with tau disaggregation (Nachman et al. 2020) and turnover (Thompson et al. 2012; Jinwal et al. 2013; Kundel et al. 2018; Baughman et al. 2018; Mok et al. 2018), and these chaperones have been shown to directly interact with several sequences in tau’s microtubule-binding repeats (Fig. 3A) (Sarkar et al. 2008; Wang et al. 2009; Jinwal et al. 2013). To better understand how these motifs bind to Hsp70s, peptides corresponding to two of these sequences ^274^KVQIINKK^281^ and ^306^VQIVYK^311^ (numbering from the 2N4R splice isoform of human tau) were examined for their ability to bind the canonical, hydrophobic groove of the SBDβ from Hsc70 (HSPA8). Fascinatingly, NMR titrations and fluorescence polarization (FP) competition studies found that only the ^274^KVQIINKK^281^ peptide bound similarly to canonical, model peptides (Taylor et al. 2018). In contrast, the ^306^VQIVYK^311^ peptide seemed to bind in a non-canonical manner that was not competitive with the model peptide. Further evidence for a non-canonical interaction came from studies focusing on tau’s KFERQ-like motifs. Briefly, KFERQ sequences are known to be required for binding to Hsp70 during chaperone-mediated autophagy (CMA) (Dice et al. 1986; Fred Dice 1990; Sahu et al. 2011; Morozova et al. 2016; Mukherjee et al. 2016). Tau is a CMA substrate and it contains two ostensible KFERQ-like sequences, ^336^QVEVK^340^ and ^347^KDRVQ^351^ (Wang et al. 2009). However, these KFERQ peptides do not compete with the model canonical peptides for binding to Hsp70’s SBD (Taylor et al. 2018), suggesting that they too bind non-canonically.

If the ^306^VQIVYK^311^, ^336^QVEVK^340^, and ^347^KDRVQ^351^ do not bind the SBDβ groove, where do they bind? While the exact details are not yet known, it is speculated that they might bind in the SBDα/lid region (Fig. 3B). Circumstantial evidence for this possibility comes from NMR titrations, in which the peptides do not interact with an SBDβ lacking the lid (Taylor et al. 2018). Furthermore, the lid and C-terminal domains are the most divergent between Hsp70 orthologs and tau is known to bind differently to the major cytosolic family members, Hsc70 and Hsp72 (Jinwal et al. 2013; Nachman et al. 2020). However, the exact site responsible for the non-canonical interactions with tau remain poorly defined and it is not clear if ^306^VQIVYK^311^, ^336^QVEVK^340^, and ^347^KDRVQ^351^ share the same interaction site(s).

More insight into non-canonical client interactions can be gleaned from recent studies using α-syn, which is known to form aggregates in Parkinson’s disease (Auluck et al. 2002; Danzer et al. 2011; Klucken et al. 2004). Like tau, disaggregation of α-syn seems to be, in part, reliant on its interaction(s) with Hsp70 (M. M. Schneider et al. 2021; Gao et al. 2015). NMR titrations have shown that Hsp70 binds at least two distinct sites on α-syn: “Site 1”—the first 12 amino acids of the protein’s N-terminus and “Site 2”—a 6 amino acid sequence centered on tyrosine 39 (Fig. 3A) (Burmann et al. 2020). While site 2 resembles a canonical sequence, site 1 does not. Consistent with this observation, site 1 binding to Hsp70 seems to be less sensitive to nucleotide, when compared to site 2 (Burmann et al. 2020). Is the non-canonical, site 1 interaction of Hsp70 with α-syn functionally important? The answer seems to be yes because a non-canonical interaction was recently reported to suppresses α-syn pathological aggregation in a nucleotide-independent fashion (Tao et al. 2021). Thus, the non-canonical interaction is not simply an “extra” contact. Although it is not yet clear where site 1 binds on Hsp70s, a C-terminal truncation demonstrated that the lid domain is required for anti-aggregation activity (Tao et al. 2021), again focusing attention on this domain as a possible site for the non-canonical contact (Fig. 3B). Interestingly, Hsp70’s lid domain is also known to interact with membranes (Morozova et al. 2016), ribosomal proteins and rRNA (Gumiero et al. 2016), and mitochondrial clients (Strub et al. 2003). Thus, these non-canonical interactions might be more widespread than previously thought.

IDPs, such as α-syn and tau, are not the only examples of clients that include non-canonical-binding sites. X-linked inhibitor of apoptosis protein (XIAP) is an important anti-cancer drug target that binds to Hsp70 with an uncharacteristically tight affinity (~ 260 nM) in vitro (Cesa et al. 2018). XIAP is composed of three baculoviral IAP repeat (BIR) domains, a ubiquitin-associated (UBA) domain, and a RING domain. Using prediction algorithms, canonical Hsp70 client–binding sites were identified in the BIR2 and BIR3 domains (Fig. 3A) and, as expected, point mutations within some of these sequences significantly weaken the interaction of XIAP with Hsp70 (Cesa et al. 2018). However, NMR studies using ^15^ N-labeled SBDβ surprisingly showed that titration with XIAP_120–356_, which contains BIR2 and BIR3 but no other domains (Fig. 3A), did not cause the expected chemical shift perturbations (CSPs) in the hydrophobic groove (Cesa et al. 2018). Competitive FP assays supported this conclusion, as the NRLLLTG peptide was unable to compete with XIAP_120–356_ for binding to Hsp70. These findings can be rationalized by the fact that although XIAP contains predicted, canonical Hsp70-binding sequences, they are present within the folded BIR2/3 domains and, thus, may not be in the linear state required to bind Hsp70s in that way. Instead, binding seems to be driven by an alternate mechanism. Concordant with this hypothesis, there was a rather surprising finding in this study—that titration of Hsp70’s NBD alone (lacking the SBDβ, lid or C-terminal domains) could abolish XIAP_120–356_ association (Cesa et al. 2018). Thus, the non-canonical interaction of Hsp70 with XIAP_120–356_ seems to require the NBD. Furthermore, this non-canonical interaction also seems to be functionally important for XIAP stability, as disrupting Hsp70 expression or activity leads to rapid degradation of XIAP in cells (Zhang et al. 2015; Cesa et al. 2018). While non-canonical client interactions with the NBD are not commonly reported in the literature, XIAP_120–356_ is not the only example. In fact, HLA-DR, an MHC II receptor protein, has also been found to interact with the NBD (Rohrer et al. 2014), and like XIAP, binds with an unusually tight affinity (~ 15 to 130 nM) (Haug et al. 2007). Further studies are needed to elucidate both the location and the role of these non-canonical client interactions for XIAP_120–356_, HLA-DR, and likely other clients.

Taken together, work on tau, α-syn and XIAP have revealed that eukaryotic Hsp70s engage in non-canonical interactions with clients. Moreover, comparing these examples reveals some shared features. For example, these interactions occur exclusively outside of SBDβ and do not compete with canonical peptides. Additionally, non-canonical interactions seem largely insensitive to the nucleotide status of Hsp70 (Tao et al. 2021; Cesa et al. 2018), which is in stark contrast to canonical clients. This nucleotide independence might be important for decoupling client interactions from the canonical ATPase cycle (see Fig. 1B) for reasons that are not yet clear. However, not all of the non-canonical interactions are the same, and client-specific differences are also notable. For XIAP, Hsp70’s NBD appears to be critical for the interaction; yet, for tau and α-syn, the lid domain (and possibly the C-terminal extension) have emerged as the most likely binding site(s). These results suggest that there could be at least two, unique non-canonical binding sites for clients on eukaryotic Hsp70s.

It is not yet clear why some clients, such as tau and α-syn, contain both canonical and non-canonical motifs. Given that these sites engage different surfaces on Hsp70 and have different nucleotide dependences, it is possible that the number and type of binding site(s) within a client may convey functional information. For example, having two types of sites may allow for multiple ways of degrading important clients through different Hsp70 complexes. One example of this logic appears to be α-syn, where mutating a KFERQ motif is sufficient to interrupt its degradation via CMA, but without affecting its global turnover (Cuervo et al. 2004). In this case, having two types of Hsp70-binding sites (canonical and non-canonical) may provide redundancy and the ability of Hsp70s to access multiple degradation pathways. It is also possible that having both canonical and non-canonical-binding sites is useful for recruiting multiple Hsp70s to a single client protein, as exemplified by the model client, hTRF1, where at least 2 Hsp70 molecules can bind simultaneously to multiple sites on a single hTRF1 polypeptide (Rosenzweig et al. 2017). Likewise, multiple points of contact have been proposed to be important for the disaggregation activity of Hsp70 systems (Szabo et al. 1994; Nillegoda et al. 2015). However, not all clients have both canonical and non-canonical-binding sites; for example, huntingtin seems to exclusively bind non-canonically to Hsp70 (Monsellier et al. 2015; Taylor et al. 2018). More work is needed to dissect the contributions of canonical and non-canonical interactions and uncover the information encoded in these combinations.

### Hsp70 nucleotide exchange factors (NEFs)

**Fig. 4:**
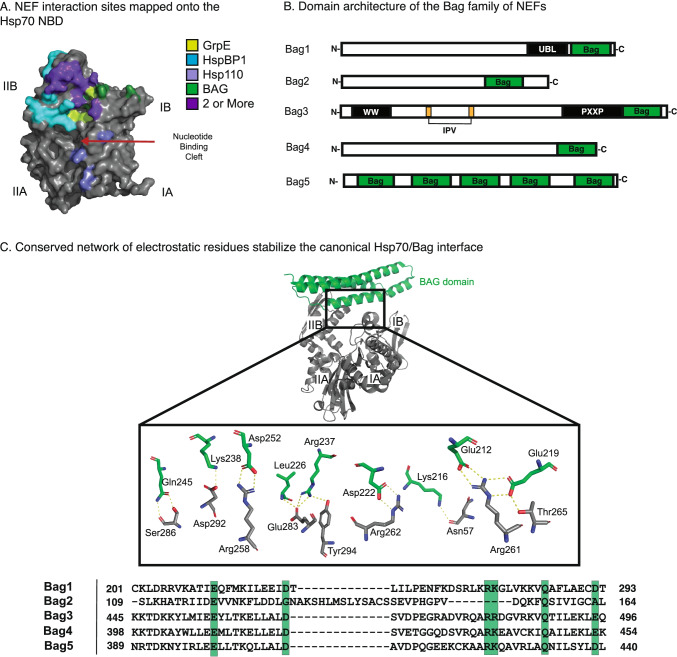
Canonical Interactions of Hsp70 NEFs are highly conserved. **A** NEF interaction sites are mapped onto the Hsc70 NBD (PDB 1HX1) and color coded by NEF. Binding sites shared by 2 or more NEFs are colored purple. **B** Domain architecture of the 5 Bag family NEFs shows significant variance outside of the C-terminal Bag domain. **C** The co-crystal structure of the human Bag1 Bag domain (green) and Hsc70 (gray; PDB 1HX1) highlight an important network of electrostatics stabilizes the PPI interface. Conservation of these residues across Bag family members is shown below (C-terminal Bag domain was used for Bag 5) from a CLUSTALW multiple sequence alignment

In eukaryotes, there are four categories of NEFs: the GrpE, Hsp110, HspBP1, and Bag families (Bracher and Verghese 2015). GrpE is most ancient of these NEFs, and its eukaryotic orthologs are localized to the mitochondria (Bracher and Verghese 2015). Despite significant variance in their amino acid sequence and three-dimensional structures, all four NEF families bind near the nucleotide-binding cleft of the NBD to accelerate ATP re-binding (Brehmer et al. 2001; Sondermann 2001; Shomura et al. 2005; Schuermann et al. 2008). We collectively refer to Hsp70’s interactions with these NEF’s domains as canonical, even though the different family members contain distinct domains and they utilize a variety of binding modes (Fig. 4A).

In addition to their canonical interaction domains, most NEFs also contain additional domains. For example, Hsp110 includes a domain that resembles Hsp70’s SBD, which binds clients (Goeckeler et al. 2008; Xu et al. 2012) and plays an essential role in protein disaggregation (Yamagishi et al. 2003; Ishihara et al. 2003; Abrams et al. 2014). The Bag proteins exemplify this modular architecture of the NEFs. The five members of this family (Bag1-5) are defined by the presence of a Bag domain at their C-terminus (Bimston et al. 1998; Shinichi Takayama et al. 1999; Gässler et al. 2001), but they differ in their other domains (Fig. 4B). For instance, Bag1 has a ubiquitin-like domain (UBL), which is hypothesized to play a role in triage of Hsp70-bound clients to the ubiquitin–proteasome system (Lüders et al. 2000; Demand et al. 2001; Alberti et al. 2002). Another prominent member of the family, Bag3, has a number of additional domains, including IPV motifs that bind to small heat shock proteins (Carra et al. 2008; Rauch et al. 2017; Guilbert et al. 2018) and a WW domain that allows it to shuttle clients to the autophagy-lysosome pathway (Merabova et al. 2015). These adapter functions of Bag NEFs appear to be functionally important because mutations in Bag3’s domains that alter its interactions with Hsp70 give rise to dilated cardiomyopathy (Homma et al. 2006; McClung et al. 2017; Judge et al. 2017; Meister-Broekema et al. 2018). Here, we briefly review the canonical interactions of the BAG family NEFs with Hsp70s before discussing the growing evidence that they also make important non-canonical contacts.

#### Canonical Hsp70/BAG interactions

In the Bag family of NEFs, the canonical interaction with Hsp70 occurs via the Bag domain. This interaction stabilizes the NBD in an “open” conformation that favors ADP dissociation and ATP re-binding (S. Takayama 1997; Bimston et al. 1998; Gässler et al. 2001; Sondermann 2001). These complexes are also relatively tight, with binding affinities in the mid-nanomolar range (Stuart et al. 1998; Rauch and Gestwicki 2014). Structural studies have shown that the Bag domains engage lobes IB and IIB of Hsp70’s NBD via a highly conserved network of electrostatic interactions (Fig. 4C) (Briknarová et al. 2001; Sondermann 2001; Arakawa et al. 2010). Indeed, mutating one of these residues to alanine is sufficient to block the interaction and interrupt Hsp70-Bag functions both in vitro (Arakawa et al. 2010; Rauch et al. 2016) and in cells (Gentilella and Khalili 2011; Colvin et al. 2014). Furthermore, the canonical interaction through the BAG domain is sufficient to promote Hsp70’s ADP release, as treatment with the Bag domains of Bag1 and Bag3 alone (without the other regions) will promote dissociation of a fluorescent nucleotide with a similar potency to the full-length proteins (~ 0.4 to 0.7 µM) (Rauch and Gestwicki 2014).

#### Non-canonical Hsp70/BAG interactions

Beyond their effects on nucleotide exchange, the NEFs have long been known to also promote client release from Hsp70. For example, addition of a NEF, such as Bag3, triggers the release of a fluorescently labeled LVEAVY peptide from the Hsp70 complex, as measured by FP assays (Rauch and Gestwicki 2014; Rauch et al. 2016). Originally, this activity was believed to be solely the result of the NEF’s impact on nucleotide cycling. In this model, favoring the ATP-bound state would eventually weaken Hsp70’s affinity for client, increase the off-rate and promote client release. However, emerging evidence suggests that client release is rapidly and actively promoted by secondary contacts with some NEFs. This is the case with both GrpE (Brehmer et al. 2004; Moro et al. 2007) and HspBP1 (Rosam et al. 2018; Gowda et al. 2018), where N-terminal regions, found outside of the canonical site of Hsp70 binding, are important for client release. Similarly, truncation studies have shown that the human Bag domain is both dispensable for rapid client release (S. Takayama 1997; Rauch et al. 2016), and that an isolated Bag domain from Bag3 (lacking the other domains) has negligible activity in client release assays (Rauch et al. 2016; Rauch and Gestwicki 2014)—even though it potently promotes nucleotide exchange within the same time and concentration regimes. Together, these findings support a model in which regions outside the NEF domains function as distinct “release domains,” stimulating client release in a way that is, at least partially, de-coupled from nucleotide exchange.

How does the non-canonical interaction promote client release? NMR studies have shown that titration of Bag3 lacking the Bag domain (Bag3-ΔBag) into a sample of ^15^ N-labeled SBD yields significant CSPs in and around the client-binding groove (Rauch et al. 2016). Thus, one simple model for the BAG family of NEFs is that the “release domains” might be pseudo-substrates, which directly compete with clients for binding to the SBD. Indeed, binding studies have shown that non-canonical interaction of Bag3-ΔBag with Hsp70 has an apparent affinity of ~ 10 μM (Rauch et al. 2016), which is similar to the affinity of many model substrates. Yet, most of the interaction between the full-length proteins must originate from the canonical interaction because the affinity for the two full-length proteins is significantly tighter (~ 3 nM in the apo state, ~ 10 nM in the ATP state, and ~ 40 nM in the ADP state) (Rauch and Gestwicki 2014). Therefore, it is possible that the canonical interaction drives initial binding, but that the secondary, non-canonical interaction, now brought in close proximity, is then important for rapidly releasing the client.

Although Hsp70’s SBDβ seems to be the primary site for binding these pseudo-substrates, early attempts to map the regions on Hsc70 required for binding to Bag1 revealed possible alternatives. Specifically, using phage display and peptide arrays, seven distinct Hsc70-derived peptides were found to associate with Bag1 (Petersen et al. 2001). While two of these peptides encompass the canonical interface with the Bag domain, three of the identified sequences are in a region of the NBD that is involved in docking the lid in the ATP-bound state (Petersen et al. 2001). Thus, it is possible that additional contacts outside the SBDβ might be involved, but their significance remains unclear.

**Fig. 5:**
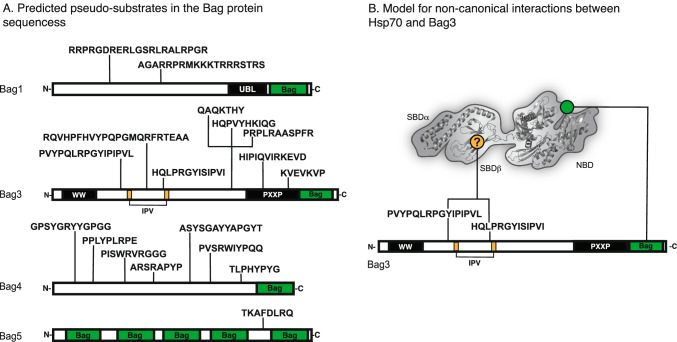
Bag family NEFs contain sequences predicted to bind Hsp70’s SBD and displace clients. **A** Sequences predicted to bind Hsp70 SBDβ within the Bag proteins. Briefly, the ChaperISM python script was used to search human Bag protein sequences in both quantitative mode (cutoff = 2.7) and qualitative mode (cutoff = 0.2). Sequences were only included if they met cutoff thresholds in both quantitative and qualitative modes, and were found outside of the Bag domain. **B** Model for how a representative BAG protein, Bag3, might use both its canonical Bag domain and its non-canonical, pseudo-substrate motif to interact with two separate sites on Hsp70. In this model, the pseudo-substrate acts as a “release domain” to promote client release from the Hsp70 complex

Likewise, the exact region of the Bag proteins that is involved in non-canonical interactions is not yet known. In HspBP1, there is a recognizable domain that resembles a substrate and it has a demonstrated interaction with SBDβ (Gowda et al. 2018). However, an equivalent site has not been identified within the Bag family and efforts to do so are complicated by the significant variance in the N-terminal sequences of the Bag proteins. Previous attempts to find pseudo-substrate sequences used the LIMBO algorithm (Van Durme et al. 2009) to computationally predict client-like sequences in both Bags 1 and 3; however, only one such motif was identified outside the Bag domain (Rauch et al. 2016). More recently, next-generation PISM-based algorithms, such as ChaperISM, have been used to re-visit this endeavor (Gutierres et al. 2019). Using the ChaperISM python script, we repeated this exercise to identify several, a priori client sequences in the N-terminal domains of Bags 1, 3, and 4 (Fig. 5A). Interestingly, two of these predicted sites in Bag3 overlap with its IPV motifs. It has been shown that IPV motifs mimic the IxI/V motifs of small heat shock proteins (sHsps) (Delbecq et al. 2012) to directly interact with sHsps (Rauch et al. 2017). If the ChaperISM predictions are accurate, then Bag3’s scaffolding activity may enable client release from both sHsps and Hsp70, perhaps assisting in client handoff between the two chaperone families. Other predicted sites are more mysterious. For instance, a potential client-like motif was also predicted in Bag5; however, this motif is located between two of its five Bag domains (Fig. 5A), casting doubt on its accessibility for interactions with the hydrophobic grove of the SBD. Regardless, we suggest that the predicted client-like sites represent starting points for better understanding non-canonical interactions between Hsp70s and Bag NEFs.

Would a single Bag protein be able to make multiple contacts with a single Hsp70? Most of the Bag proteins contain stretches of disorder; AlphaFold predicts substantial disorder outside of the Bag domain for Bags 1, 3, and 4 (Jumper et al. 2021; Varadi et al. 2022). This prediction is supported by light scattering experiments, which have shown that Bag1 has an overall, elongated shape, with a hydrodynamic radius of ~ 3.3 nm (Stuart et al. 1998). This degree of disorder may allow the Bag domain to make contact with the NBD, while still permitting the N-terminal region of Bag1 to make contact with SBDβ (Fig. 5B). The alternative is that multivalent PPIs in the BAG-Hsp70 complex involve inter-molecular, bridging contacts (i.e., one Bag protein binding two Hsp70s). The contributions of these putative binding modes and their functional importance remain unclear.

### J-domain proteins

JDPs comprise a family of important Hsp70 co-chaperones. The defining feature of these proteins is the presence of a conserved J-domain, which is named from the *Escherichia coli* ortholog, DnaJ (Kampinga and Craig 2010). This J-domain directly binds to the NBD and linker regions in the ATP state to accelerate hydrolysis of ATP to ADP, “trapping” bound clients at the SBDβ (Minami et al. 1996; Laufen et al. 1999; Kampinga and Craig 2010). The JDP family is further sub-divided into three classes (classes A, B, and C), which vary except for the presence of the J-domain. Here, we will focus on the class B proteins, which contain an N-terminal J-domain, a Gly/Phe-rich (G/F) linker, two C-terminal domains (CTDI and CTDII), and, sometimes, a dimerization domain (DD) (or oligomerization sequence) at the extreme C-terminus (Kampinga and Craig 2010). The canonical interaction between Hsp70s and class B JDPs occurs through the conserved J-domain, while other domains are involved in additional contacts; for example, the G/F motif autoinhibits the J-domain (Karamanos et al. 2019; Faust et al. 2020), the DD mediates self-assembly (Sha et al. 2000; Li et al. 2003; Hu et al. 2008; Suzuki et al. 2010; Jiang et al. 2019), and the CTDs bind clients (Li et al. 2003; Sha et al. 2000; Jiang et al. 2019). Among the co-chaperone families, the JDPs have been most broadly linked to disease. For example, dysregulation of JDP expression or activity has pathological consequences in diseases including cancer (Chen et al. 2002; Isomoto et al. 2003; Kanazawa et al. 2003; Syken et al. 1999; Tang et al. 2005; Tsai et al. 2006), viral infection (Campbell et al. 1997; Genevaux et al. 2003; Kelley and Georgopoulos 1997) and neurodegenerative disease (Hageman et al. 2010; Månsson et al. 2014; Kakkar et al. 2016, 2016; Mok et al. 2018; Westhoff et al. 2005; Claeys et al. 2010; Chen et al. 2016). Hence, much attention has been directed to the PPIs between Hsp70s and JDPs as a key axis to understanding proteostasis in disease.

#### Canonical Hsp70/JDP interactions

**Fig. 6:**
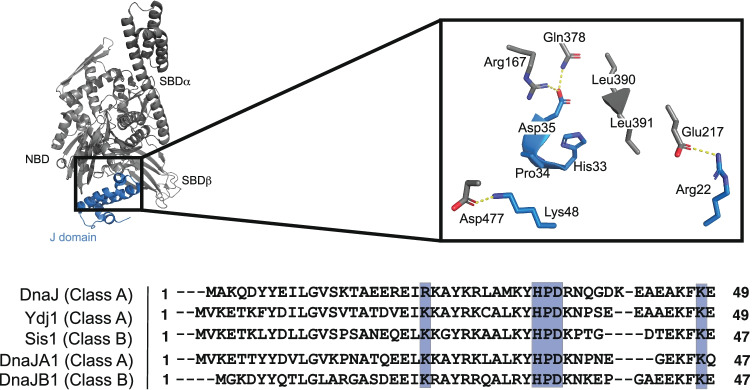
The canonical interaction of Hsp70s with JDPs is mediated by three major contacts within the J-domain, as shown in a co-crystal structure of the *E. coli* DnaK/DnaJ system (PDB 5NRO). The conservation of those residues across Hsp70 orthologs in *E. coli* (DnaJ), yeast (Ydj1 and Sis1), and humans (DnaJA1 and DnaJB1) is shown from a CLUSTALW multiple sequence alignment

Much of our knowledge on the canonical-binding interaction between Hsp70 and JDPs comes from studies of the bacterial proteins: DnaK and DnaJ. Briefly, this work has revealed that the J-domain interacts with Hsp70’s NBD using two sets of complementary electrostatic surfaces, as well as a third contact in which the invariable HPD motif between helices II and III contacts Hsp70’s interdomain linker (Fig. 6) (Ungewickell et al. 1997; Greene et al. 1998; Matthias P. Mayer et al. 1999; Suh et al. 1999; Ahmad et al. 2011; J. Jiang et al. 2007; Kityk et al. 2018). Mutations in these sites hinder association with Hsp70 and block the stimulation of ATP turnover (Kityk et al. 2018; Tomiczek et al. 2020), supporting their functional importance. The affinity of the J-domain for Hsp70s has been measured using a variety of approaches and is variably estimated to be between 0.07 and 0.54 _μ_M (Suh et al. 1998; Suh et al. 1999) or 5 to 10 _μ_M (Greene et al. 1998). The disparity between these values might arise from differences in experimental conditions and the question deserves to be re-explored. Regardless, this canonical interaction is likely the major contributor to the overall interaction affinity, because the full-length proteins bind with a *K*_*d*_ ~ 3.6 µM (Matthias P. Mayer et al. 1999). It should be noted that the mechanism by which the G/F region regulates the J-domain interaction the Hsp70 has only recently been uncovered (Faust et al. 2020), so it will be important to re-evaluate the overall interaction affinities and the contributions of the J-domain contact within that context. Finally, it is worth noting that there are some variations in canonical J-domain recognition by specialized Hsp70 systems (Schilke et al. 2006; Ciesielski et al. 2012, 1; Uhrigshardt et al. 2010; Delewski et al. 2016). All together, it is clear that the J-domain contact with Hsp70s is ancient, well conserved, and essential for stimulating ATPase activity.

#### Non-canonical Hsp70/JDP interactions

**Fig. 7:**
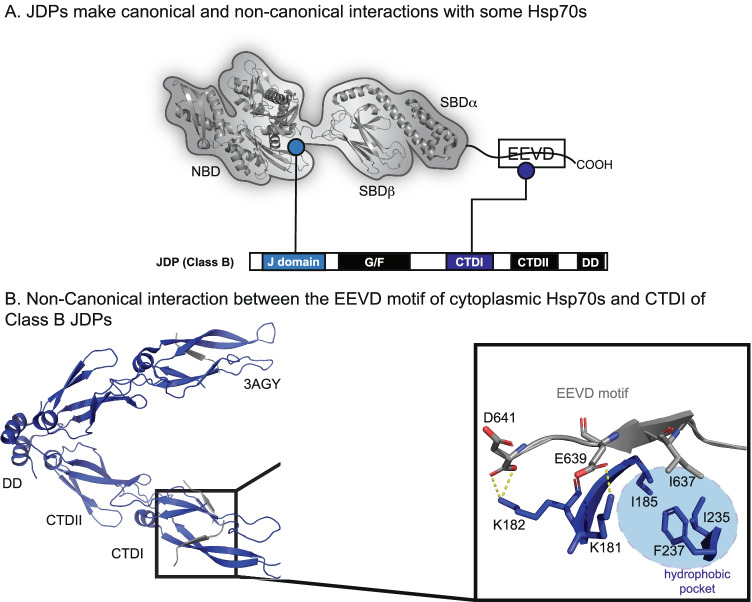
JDPs and Hsp70 interact using multiple sites. (A) The conserved J-domain binds to the NBD and linker of Hsp70s (see Fig. 6). In addition, Class B JDPs also bind to the EEVD motif present in cytoplasmic Hsp70s. (B) Co-crystal structure of an Hsp70 derived EEVD motif peptide (gray) bound to CTDI of human DnaJB1 (blue; PDB 3AGY), highlighting the key residues responsible for complex formation

Hints of a non-canonical interaction between JDPs and eukaryotic Hsp70s arose in genetic studies, which showed that mutation or deletion of an EEVD motif, found at the extreme C-terminus of cytosolic Hsp70s, blocked Hsp70-JDP collaboration in cells (Lopez-Buesa et al. 1998). This finding was also observed in vitro, as the Class B JDP, DnaJB1, was shown to interact with immobilized, full-length GST-Hsp70, but not with a variant lacking the C-terminal EEVD (Freeman et al. 1995; Demand et al. 1998). These data were initially puzzling because the EEVD motif is located far from the site of the canonical J-domain interaction. However, since then, a number of structural and biochemical studies have refined our knowledge of this non-canonical interaction. Specifically, the EEVD motif has been found to interact with beta-sheets in CTDI of Class B JDPs (Fig. 7A) (Sha et al. 2000; Li et al. 2006; Suzuki et al. 2010). Co-structures of complexes formed between EEVD peptides and CTDI of DnaJB1, solved by x-ray crystallography, revealed that the PPI was mediated by both an electrostatic interaction, between Lys182 and the C-terminal carboxylate, and interactions of Ile637 of the Hsp70 EEVD motif with a hydrophobic pocket (Fig. 7B) (Suzuki et al. 2010). We recently confirmed the importance of these contacts, using truncations and mutations of Hsp70-derived EEVD peptides (Johnson et al. 2022). Furthermore, NMR experiments showed that the EEVD motif exhibits selectivity for binding CTDI over CTDII in the class B JDPs of humans and yeast (Jiang et al. 2019; Faust et al. 2020). As mentioned above, this non-canonical PPI has also been revealed to serve a crucial role in regulating the canonical Hsp70-binding interface of the J-domain (Faust et al. 2020; Jiang et al. 2019; Karamanos et al. 2019). Specifically, binding of the EEVD motif at CTDI produces long-range conformational change that disrupts an autoinhibitory helix near the J-domain, liberating the canonical-binding site (Faust et al. 2020). Thus, for the class B JDPs, the non-canonical and canonical PPIs are in close communication.

Intriguingly, the EEVD motif in Hsp70s is also the primary site for interacting with the TPR family of co-chaperones (Allan and Ratajczak 2011; Weber et al. 2020), and early evidence suggested that this region was subject to direct competition between the two co-chaperone families (Demand et al. 1998). In recent studies, this competition was directly measured, showing that both DnaJB1 and DnaJB4 can partially block the function of a complex between Hsp70 and the TPR protein, CHIP, whereas CHIP can block folding by the Hsp70-DnaJB4 complex in vitro (Stankiewicz et al. 2010; Johnson et al. 2022). Notably, the EEVD does not bind in an identical configuration in the CTDI and TPR domains; it is linear and extended in the CTDI-bound complex, but “bent” in the TPR-bound complex. Moreover, the CTDI exhibits a much higher tolerance for mutations in the EEVD motif; for example, aromatic residues at Hsp70 residue 637 significantly hinder TPR binding while peptides with Phe or Tyr at residue 637 bind comparably to the wild-type Ile637 peptide (Johnson et al. 2022). Taken together, these data suggest that class B JDPs and TPRs evolved to engage in competition for binding at the EEVD motif, tuning Hsp70’s activity.

In addition to its ability to bind Hsp70, CTDI of class B JDPs, along with CTDII, directly bind client proteins. NMR titration experiments have shown that the Class B JDPs bind clients via CTDI, which shows selectivity for a subset of non-native client sequences (Jiang et al. 2019; Faust et al. 2020; Lee et al. 2002). Thus, the EEVD motif and client proteins must, presumably, directly compete for binding CTD I. This feature supports the possibility that the EEVD regulates the handoff of clients from Class B JDPs to Hsp70s (Sha et al. 2000; Jiang et al. 2019). In this model, clients first bind the CTDI and are then released, in part, by competition with the EEVD motif, presumably for delivery to Hsp70’s SBDβ. While this speculative hypothesis requires additional study, it is clear that the non-canonical interaction, in addition to the classic J-domain interaction, is critical to coordination of at least a subset of Hsp70-client complexes.

Multiple sites of contact between the class B JDPs and Hsp70s might serve another purpose. Specifically, it is known that these proteins, plus Class A JDPs and Hsp110, can form a complex that is able to disaggregate protein deposits (Nillegoda et al. 2015; Nillegoda et al. 2018). Having two sites of binding might allow the Hsp70s and JDPs to coordinate via intra- and inter-molecular contacts, favoring the geometry and orientation of the components within the machinery to carry out this complex disaggregation function.

## Discussion

The proteome complexity of organisms has expanded significantly throughout evolution. Thus, one might postulate that, as the number of potential client proteins, proteoforms and PPIs expanded (Bludau and Aebersold 2020), the molecular chaperone machinery needed to co-evolve. Indeed, eukaryotes possess a greater number of both Hsp70 and co-chaperone genes compared to prokaryotes (Kominek et al. 2013) and these “newer” chaperones are associated with specialized processes, such as clathrin uncoating (Ungewickell et al. 1997), protein maturation (Vembar et al. 2010; Shen and Hendershot 2005), folding/stabilization (Arndt et al. 2005), and degradation (Alberti et al. 2002; Carra et al. 2008; Gamerdinger et al. 2011; Bhattacharya et al. 2020). Concurrently, the increased diversity of sub-cellular compartments in eukaryotes has demanded expansion of chaperone systems into those spaces. For Hsp70, which works so closely with co-chaperones and clients, this expansion likely placed increased demands on its ability to form a diverse array of functional complexes. Thus, while the canonical interactions, including Hsp70 binding to the J-domain, NEF domains and hydrophobic client peptides, have remained as crucial drivers of chaperone activity in eukaryotes, additional, non-canonical interactions have also emerged.

In some cases, non-canonical interactions with Hsp70 have been found to be necessary and sufficient to drive chaperone functions, such as autophagic degradation (for clients) (Cuervo et al. 2004), client release (for NEFs) (Moro et al. 2007; Rauch et al. 2016; Gowda et al. 2018), and J-domain activation (for Class B JDPs) (Faust et al. 2020). Thus, one way to think about non-canonical interactions is that they are not “extra.” Rather, they customize and/or diversify the Hsp70 interactome and, therefore, expand Hsp70’s functions. These binding sites might also exert more control over the chaperone’s decision-making. For example, the secondary contact between the EEVD motifs of eukaryotic Hsp70’s and CTDI of class B JDPs allows the complex to be tuned by competition with both clients (Jiang et al. 2019; Faust et al. 2020) and TPR proteins (Demand et al. 1998; Stankiewicz et al. 2010; Johnson et al. 2022). In this case, cells might adjust their relative levels of TPR proteins, for example, to change which co-chaperones contact is favored and re-direct clients to specific fates. Thus, by using multivalent and modular PPIs, eukaryotic Hsp70 systems might have evolved more precise control.

Yet, our knowledge of non-canonical contacts and their roles in the Hsp70 complexes is far from complete. While some mechanistic information has been gleaned (and reviewed here), there are many important questions remaining. First, most studies of non-canonical interactions have used 1 or 2 representative co-chaperones within the families (e.g., Bag1 and Bag3 NEFs), so generality to other members is not clear. Additionally, few studies have asked whether secondary contacts impact the overall binding kinetics. This is an important question because multivalent interactions often enhance the dwell time of complexes, through slower off-rates and higher occupancy (Mammen et al. 1998; Gestwicki et al. 2000; Gestwicki et al. 2002). It was recently shown that tighter binding of tau by Hsp70 favors its degradation (Young et al. 2016); hence, the secondary contacts could be important in controlling dwell time. Another major gap in knowledge is that few structural details are available for most non-canonical interactions. Where do the non-canonical client sequences, such as KFERQ, bind on Hsp70? How do the “release domains” of Bag or HspBP1 NEFs work to dislodge clients? Finally, another outstanding question is how secondary binding sites contribute to cellular proteostasis. The majority of knowledge on non-canonical PPIs of Hsp70s comes from in vitro studies; thus, it will be important to conduct further studies in cells and animals to understand their role(s) in a cellular context. Mutations that disrupt non-canonical interactions, while sparing the canonical ones, will be particularly powerful.

Given the key roles for Hsp70 complexes in disease, its PPIs are potential targets for chemical probes (Balch et al. 2008; Gestwicki and Shao 2019). While pan-inhibitors of all Hsp70 functions would likely be toxic, it seems logical to predict that targeting a subset of its PPIs would be safer. To date, efforts to create such chemical probes and therapeutics have largely focused on molecules that disrupt canonical interactions with the J-domain (Wisén et al. 2010) and the Bag domain (Shao et al. 2018), as well as the EEVD motif (Vasko et al. 2010; Zaiter et al. 2019; Ravalin et al. 2019). Accordingly, we speculate that an in-depth understanding of non-canonical contacts could open new possibilities to create molecules that more finely tune Hsp70-mediated proteostasis as a way to treat diseases such as cancer and neurodegeneration.
